# A Nickel Ain't Worth a Dime Anymore: The Illusion of Money and the Rapid Encoding of Its True Value

**DOI:** 10.1371/journal.pone.0055025

**Published:** 2013-01-30

**Authors:** Rongjun Yu, Yi Huang

**Affiliations:** School of Psychology and Center for Studies of Psychological Application, South China Normal University, Guangzhou, China; University of Missouri-Kansas City, United States of America

## Abstract

People often evaluate money based on its face value and overlook its real purchasing power, known as the money illusion. For example, the same 100 Chinese Yuan can buy many more goods in Tibet than in Beijing, but such difference in buying power is usually underestimated. Using event related potential combined with a gambling task, we sought to investigate the encoding of both the real value and the face value of money in the human brain. We found that the self-reported pleasantness of outcomes was modulated by both values. The feedback related negativity (FRN), which peaks around 250ms after feedback and is believed to be generated in the anterior cingulate cortex (ACC), was only modulated by the true value but not the face value of money. We conclude that the real value of money is rapidly encoded in the human brain even when participants exhibit the money illusion at the behavioral level.

## Introduction

Money is a commodity accepted by general consent as a medium of economic exchange. Its real value lies only on its buying power in economic transactions, which is the number of goods/services that can be purchased with a unit of currency. The face value of money may still remain the same when its real value changes dramatically. For example, 100 dollars in 1912 may worth much more than 100 dollars in 2012. If you had taken one dollar to a store in 1912, you would have been able to buy a greater number of items than you would in 2012, indicating that you would have had a greater purchasing power in 1912. Similarly, the same 100 Chinese Yuan can buy much more goods in Tibet (a city with low price level) than in Beijing (a city with high price level), indicating that the purchasing power in Tibet is higher than that in Beijing. However, people usually evaluate money based on its face value and ignore its real purchasing power. People are generally not sensitive to variations in inflation and prices and treat 100 dollars in different situations similarly. The way human decisions are frequently affected by the nominal rather than the real value of money is referred as the money illusion [1]–[5].

However, the presence of money illusion is only inferred indirectly from its effects on behavior. Much of the evidence that has been put forward in favor of money illusion can also be explained by alternative rational theories [6]. Since previous behavior studies could not directly observe the cognitive processes that give rise to money illusion, researchers are skeptical about the notion of money illusion [7]. A previous study using functional magnetic resonance imaging (fMRI) has shown that brain activity in the ventromedial prefrontal cortex (vmPFC) in response to monetary prizes increases with nominal changes that have no consequence for participants’ real purchasing power, exhibiting money illusion at the neural level [5].

Although this study demonstrates that certain brain regions encode nominal representation rather than the real value of money, it is still unclear whether the true value of money is encoded or not and how. It is unknown whether the true value of money is encoded in certain brain regions but such signals are overridden by the face value signals or the true value has never been registered in the brain?

Here, we use event related potential combined with a gambling task to investigate the encoding of both real value and the face value of money in the human brain. There were two conditions: In the expensive price condition incomes and catalog prices were higher than in the second, cheap price condition. Thus, the face value was identical in the expensive and the cheap price conditions but real purchasing power differed. For low magnitude in cheap condition and high magnitude in expensive condition, the true value was identical but the face value differed. We examined the feedback-related negativity (FRN) and the P300, two even-related potential (ERP) components implicated in reward processing [8]. The FRN, which peaks at around 250 ms after feedback onset, is maximal at frontal-central scalp electrode sites and is most likely generated at the ACC [8], [9]. The FRN has been found to be differentially sensitive to unfavorable outcomes such as incorrect responses and monetary losses [8], [9]. The P300, which is the most positive peak in the 200–600ms time window post-onset of feedback, has also been shown to be sensitive to the valence of reward [10], [11]. As such, the FRN and the P300 can provide useful markers for investigating the time-course of reward processing.

## Materials and Methods

### Participants

Nineteen healthy, right-handed participants (9 male; mean age ± SD, 21.21±1.75 years) participated in return for payment. All the participants were right- handed, and had normal or corrected-to-normal vision, and were screened for neurological or psychiatric disorders. The study was approved by the Academic Committee of the School of Psychology at South China Normal University. All participants gave written, informed consent. They were informed of their right to discontinue participation at any time.

### Stimuli

1120 landscape images were downloaded online, carefully divided into 560 pairs. In a pilot study, for each image pair, ten individuals were asked to choose one image, which they think most people would choose. To minimize the predictability of one’ choice in each image pair, we selected image pairs in which one particular image was selected by half of the ten individuals. These image pairs were used in the experiment without replacement.

### Experimental Paradigm

At the beginning of each trial, participants were first presented with the price condition for that trial: “cheap price” or “expensive price” for 2 seconds. To this end participants did not earn their income in cash but had to spend it on a large but fixed menu of items. We created 2 catalogs with 40 items including books, CDs, DVDs, sports articles, cosmetics, and consumer electronics. The catalogs were identical with the exception that all prices were 50% higher in expensive price condition than in cheap price condition. Prices in the catalog with “cheap price” ranged from ¥1.4 to ¥24.6.

**Figure 1:**
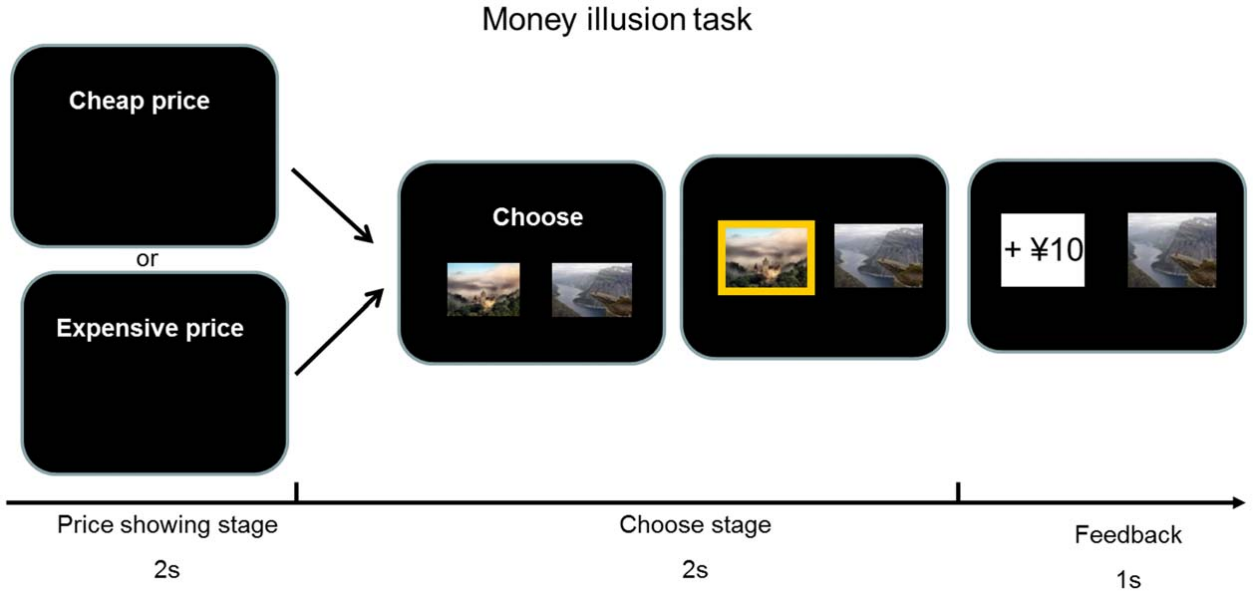
Experimental task design. At the beginning of each trial, the cheap price or the expensive price context information was shown. Then participants performed a simple gambling game in which they win or lose money on the basis of unpredictable outcomes. Participants chose one gambling card from the two and received winning or losing feedback.

Then two photos of landscapes were presented and participants were required to select one of them by pressing the left or right keys in keyboard within 2 seconds. Participants were told that one photo of landscape was associated with a win and the other photo of landscape was associated with a loss. They had to guess which one was associated with a win. The selected photo was highlighted. Then the amount of winning or losing (from ¥2.8 to ¥49.2) associated with the chosen card was shown for 1 second. Participants were informed about the range of winning or losing magnitude (from ¥2.8 to ¥49.2). Unknown to the participants, outcomes were predetermined and fully randomized across conditions. The next trial began 1 second after the offset of the feedback (Figure 1). The experiment consisted of 4 blocks of 70 trials each.

Before participants began the task, they read the instructions for the experiment and were given the opportunity to familiarize themselves with the 2 catalogs. Then they were asked to answer several control questions to make sure that they had understood the difference between the 2 catalogs; e.g., participants were asked how much an item with price *p* in the cheap price condition would cost in the expensive price condition. They were told that their performance in the task determined how much they would be awarded and how many things they could buy at the end of the experiment. All the participants received a base payment of 30 yuan (about 5 US dollar).

After the electroencephalogram (EEG) session, participants were required to indicate their feelings (pleasantness and surprise) about the eight types of outcomes (i.e. losing/winning ¥10/¥20 in cheap/expensive price condition) they experienced in the experiment on a 10-point Likert scale. After completion of the experiment, five trials in each catalog (5 trials in expensive condition and 5 trials in cheap condition) were randomly selected for actual payment. The accumulated total winnings were used for participants to buy things only in the corresponding catalog. For example, the accumulated total winnings of five trials in cheap condition were used for participants to buy things in the low price catalogue, whereas the accumulated total winnings of five trials in expensive condition were used to buy things in the high price catalogue. Participants did not earn their income in cash but had to spend it to buy things. Before the experiment, participants were clearly informed of these rules and were familiar with the two catalogues. They were not endowed with initial money and if the accumulated total winnings of five trials were negative or smaller than the cheapest price in the corresponding catalog, they cannot buy any item in that catalog. Thus, losing money in our experiment meant losing the opportunity to buy items in the respective catalog rather than losing out of pocket money.

### ERP Recording and Analysis

Standard ERP recording and analysis were applied. EEGs were recorded from 64 scalp sites using Ag/AgCl electrodes embedded in an elastic cap (NeuroScan Inc., USA) according to the international 10–20 system, with the reference to the right mastoid. Eye blinks were recorded from electrodes located above and below the left eye. The horizontal electro-oculogram (EOG) was recorded from electrodes placed 1.5 cm lateral to the left and right external canthi. The EEGs were re-referenced ofine to the linked mastoids. All electrode impedances were maintained below 5 kΩ. The EEG and EOG were amplified using a 0.05–70 Hz bandpass and continuously sampled at 500 Hz/channel for off-line analysis.

Ocular artifacts were corrected with an eye-movement correction algorithm [12]. The EEG data were re-referenced offline to linked-mastoid electrodes by subtracting 50% of the signal in the right mastoid electrode from the signal in each channel. All trials in which EEG voltages exceeded a threshold of ±70 µV during the recoding epoch were excluded from analysis. The EEG data were filtered using a 20 Hz low-pass (24 dB octave roll off), and were baseline corrected by subtracting from each sample the average activity of that channel during the baseline period. Epochs of 800 ms (with 200 ms pre-stimulus baseline) EEG from each electrode were time-locked to the onset of feedback and were sorted by experimental conditions.

According to visual inspection of ERP waveforms, the FRN for both win and loss trials were measured as the mean amplitudes in the time window of 200 to 350 ms post-onset of the feedback. The peak value of the P300 was detected as the most positive value in the 300 to 500 ms post-stimulus time window at electrode Pz. We focused on the FRN responses on the anterior frontal midline electrodes (Fz) and the P300 responses on the posterior midline electrodes (Pz), since the FRN and P300 effects were the largest on these electrodes, respectively.

## Results

### Behavioral Results

**Figure 2:**
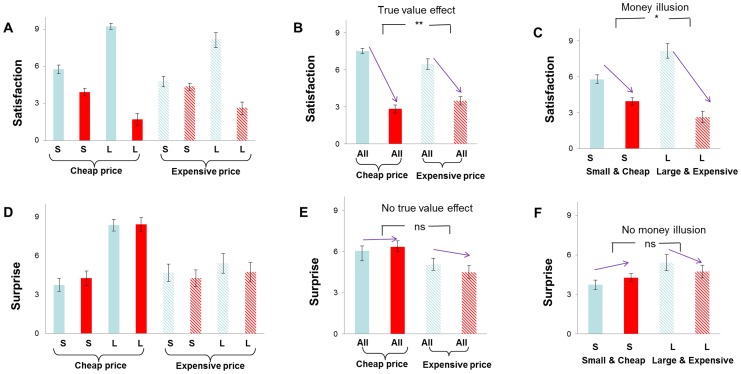
Post-experiment subjective ratings of feeling. The self-reporting satisfaction scores (mean ± SE) for the eight experimental conditions were shown in (A). The difference in satisfaction between losses (in red) and wins (in blue) was larger when the price was cheap versus expensive (B). The difference in satisfaction between losses and wins was larger for large magnitude in expensive context than for small magnitude in cheap context, showing money illusion (C). The self-reported surprise scores (mean ± SE) for the eight conditions were shown in (D). The experienced surprise was not modulated by the true value of money (E) or the face value of money (F). S: small magnitude; L: large magnitude; All: across small and large magnitude. ^*^ p<0.05, ^**^ p<0.001.

Post experiment ratings for 8 conditions were plotted in Figure 2. For the self-reported pleasantness, repeated-measures ANOVA using price (cheap vs. expensive), magnitude (small vs. large), and feedback valence (loss vs. win) as independent factors found a significant main effect of magnitude F(1,18) = 8.48, p<0.01 and feedback valence, F(1,18) = 91.99, p<0.001. The interaction between price and valence was significant, F(1,18) = 7.32, p = 0.014, suggesting that true value influences outcome evaluation. The satisfaction difference between winning and losing was larger in cheap condition (mean ± SD, 4.68±1.29) than in expensive condition (mean ± SD, 2.97±2.86). There was also a significant interaction between magnitude and valence, F(1,18) = 60.58, p<0.001, suggesting that nominal value modulates self-reported pleasantness of outcomes. No other effects was significant, p>0.2.

**Figure 3:**
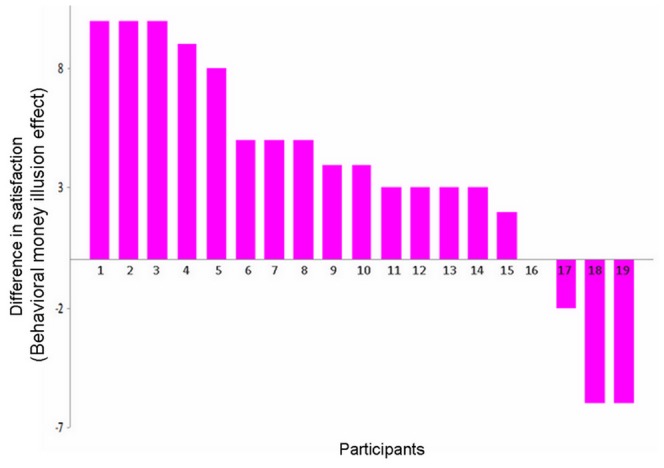
The money illusion effect for each participant. The behavioral money illusion effect for 19 participants (numerically ordered). The y axis represents the differences in satisfaction (difference between win and loss for large magnitude in expensive context - difference between win and loss for small magnitude in cheap context).

To test whether there was a money illusion effect on satisfaction, we compared winning or losing small magnitude in cheap price context with winning or losing the large magnitude in expensive price context (Figure 2C). These two contexts differed in nominal terms but were identical in real buying power. There was a significant main effect of valence, F(1,18) = 55.89, p<0.001, and a significant interaction between valence and magnitude, F(1,18) = 11.32, p<0.005. The main effect of reward magnitude was not significant, F(1,18) = 3.54, p = 0.08. The satisfaction difference between winning and losing was larger for high magnitude condition (mean ± SD, 5.53±4.33) than for low magnitude condition (mean ± SD, 1.84±1.38), indicating a money illusion at the behavioral level. Indeed, post hoc T-tests showed that participants felt more pleasant for winning large magnitude reward in high-price condition (mean ± SD, 8.16±0.60) than for winning small magnitude reward in low-price condition (mean ± SD, 5.79±0.36), t^18^ = 3.65, p<0.005, although they were identical in real terms (Figure 2B). Similarly, participants also felt more unpleasant (mean ± SD, 2.63±0.49) for losing large magnitude and high-price reward than for losing small magnitude and low-price reward (mean ± SD, 3.95±0.31), t^18^ = 2.28, p<0.05. The money illusion effect for each participant was shown in Figure 3. Most of the participants (15 out of 19 subjects) exhibited positive money illusion as shown in their satisfaction ratings. The mean money illusion (difference in satisfaction) was 3.68±4.77, ranging from −6 to 10. A previous study using a questionnaire measure of money illusion only found 8 out of 18 subjects showed positive money illusion (see their Fig. 2C) [5], suggesting that our task based measure is more sensitive than questionnaire measure in detecting behavioral money illusion.

For the self-reported surprise in response to outcomes (Figure 2 D–F), repeated-measures ANOVA revealed a significant main effect of price, F(1,18) = 13.31, p<0.005, a significant main effect of magnitude, F(1,18) = 42.54, p<0.001, and a significant interaction between the two factors, F(1,18) = 6.69, p = 0.019. No effects involving feedback valence was significant, p>0.1, suggesting that the pleasantness patterns cannot simply be explained by surprise.

### ERP Results

**Figure 4:**
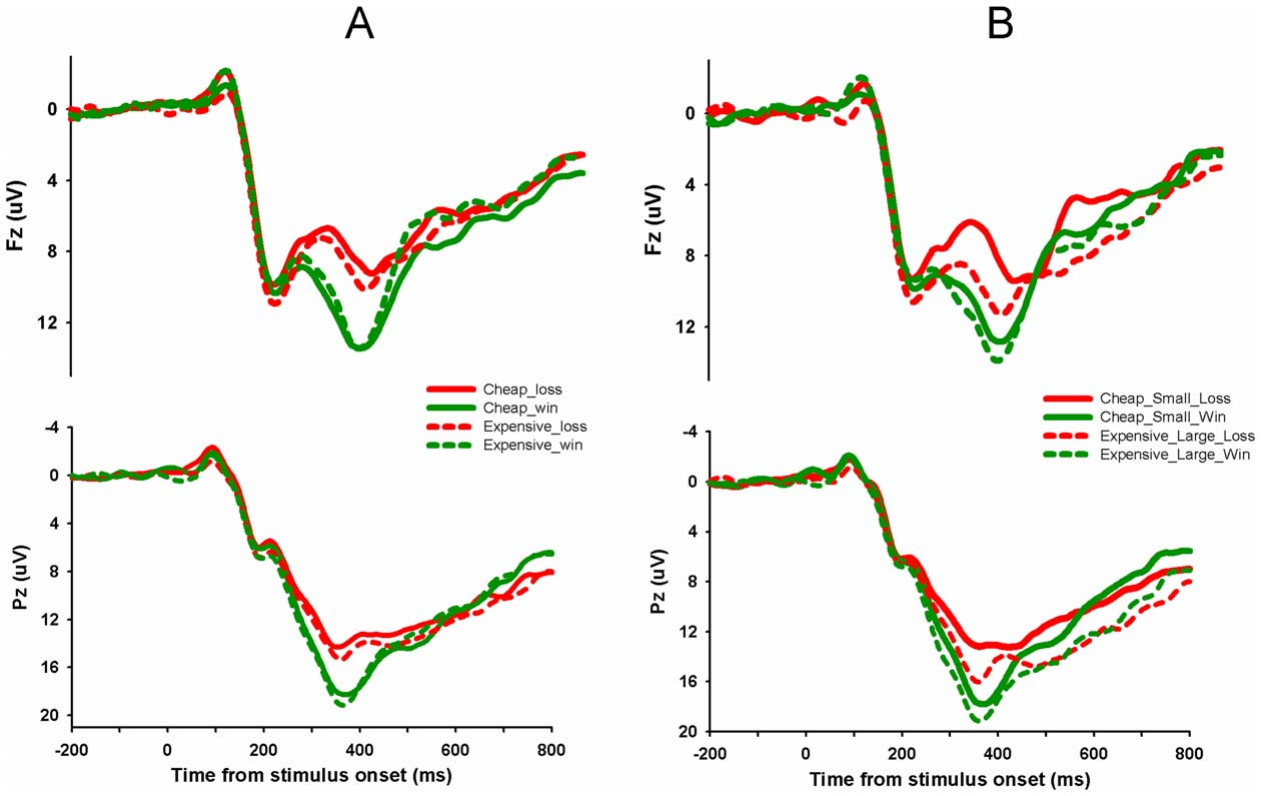
The ERP grand-average waveforms. (A) Grand-average waveforms at channel Fz and Pz for conditions that differed in real value but were identical in face value of money. (B) Grand-average waveforms at channel Fz and Pz for conditions that differed in face value but were identical in real value of money. Cheap_Loss: losing in cheap price context across magnitude; Cheap_Win: winning in cheap price context across magnitude; Expensive_Loss: losing in expensive price context across magnitude; Expensive_Win: winning in expensive context across magnitude; Cheap_Small_Loss: losing the small magnitude in cheap condition; Cheap_Small_Win: winning the small magnitude in cheap condition; Expensive_Small_Loss: losing the large magnitude in expensive condition; Expensive_Small_Win: winning the large magnitude in expensive condition.

**Figure 5:**
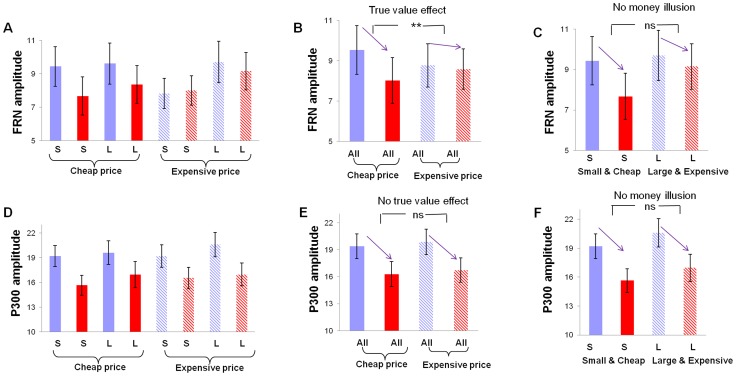
The amplitudes of FRN and P300. The FRN amplitudes (mean ± SE, in µV) for the eight experimental conditions were shown in (A). The difference in FRN amplitude between losses (in red) and wins (in blue) was larger when the price was cheap versus expensive, suggesting that the FRN is sensitive to the true value of money (B). The FRN effect (losses minus wins) was similar between large magnitude in expensive context and small magnitude in cheap context (C). The P300 amplitudes (mean ± SE, in µV) for the eight conditions were shown in (D). No effect of true value (E) and face value (F) on P300 was found. S: small magnitude; L: large magnitude; All: across small and large magnitude. ^*^ p<0.005.

For the FRN amplitude (Figure 4 and Figure 5A), repeated-measures ANOVA using price (cheap vs. expensive), magnitude (small vs. large), and feedback valence (loss vs. win) as independent factors found a significant main effect of magnitude, F(1,18) = 10.59, p<0.005 and a significant main effect of feedback valence, F(1,18) = 7.34, p<0.05. Importantly, there was a significant interaction between price and valence, F(1,18) = 6.91, p<0.05, suggesting that the FRN was sensitive to the buying power. The FRN effect (losses minus wins) was significant larger in cheap price condition (mean ± SD, −1.51±0.30 µV) than in expensive condition (mean ± SD, −0.19±0.48 µV), demonstrating a diminished sensitivity to outcome valence when the true value of money is low (Figure 5B). No other effects was significant, p>0.2.

**Figure 6:**
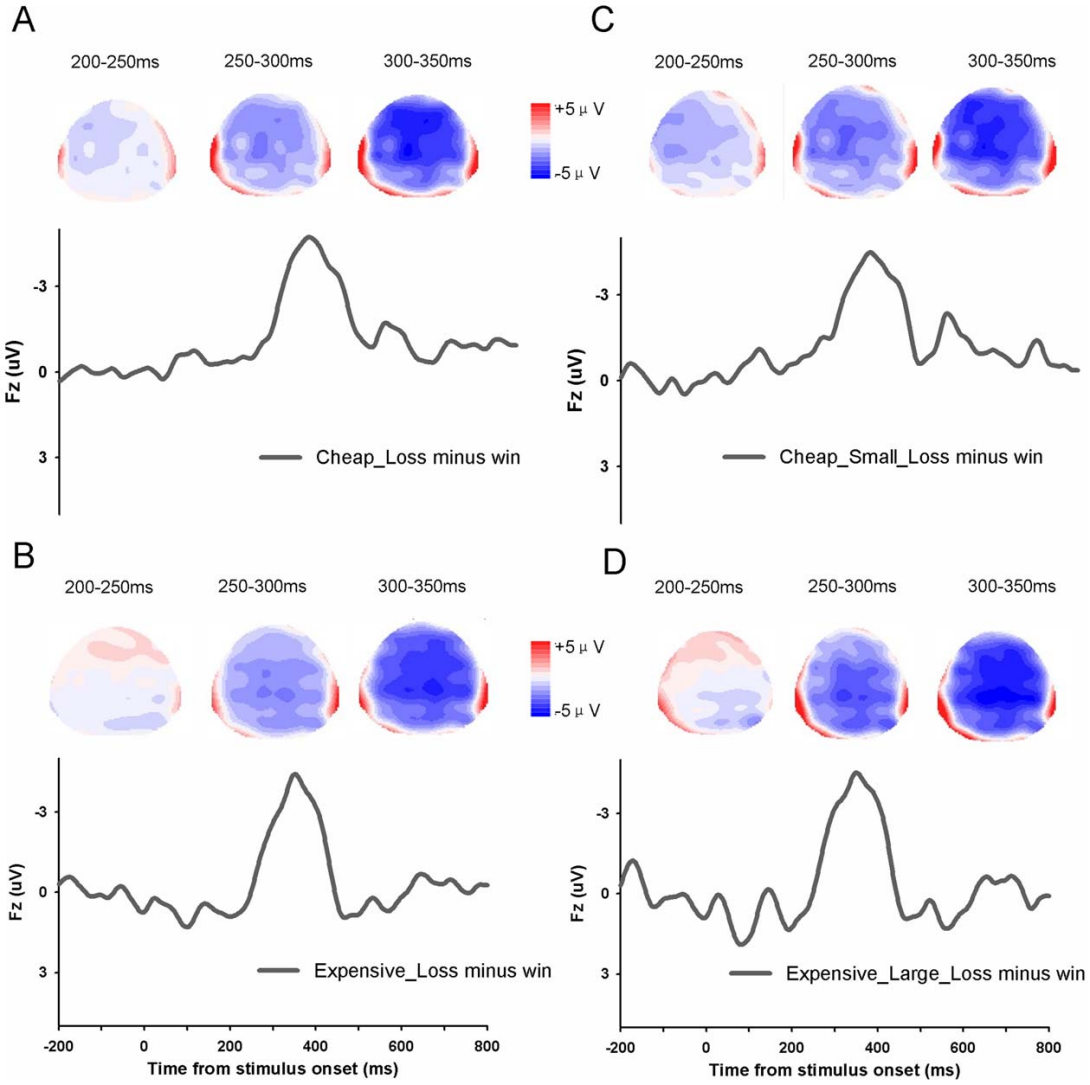
The difference waveforms and topographical maps. (A) Difference waveform (loss-win) and maps of cheap condition. (B) Difference waveform (loss-win) and topographical maps in expensive condition. (C) Difference waveform (loss-win) and topographical maps in small magnitude and cheap condition. (D) Difference waveform (loss-win) and topographical maps in large magnitude and expensive condition.

To test whether the FRN shows money illusion effect, we first compared winning or losing small magnitude in cheap price context with winning or losing the large magnitude in expensive price context (Figure 5C). These two contexts differed in nominal terms but were identical in real terms. If the FRN exhibits money illusion, we would expect larger FRN effect (losing minus winning) for large_expensive condition than for small_cheap condition. There was a marginal significant main effect of nominal terms (small_cheap vs. large_expensive), F(1,18) = 4.18, p = 0.056 and a significant main effect of valence, F(1,18) = 32.73, p<0.001. But there was no significant interaction between the two conditions, F(1,18)<1, suggesting that the FRN effect did not differ when only the face value was changed. Actually, although they were not statistically significant, the FRN effect (losses minus wins) was even larger in small_cheap condition (−2.74±0.44µV) than in large_expensive condition (−2.06±0.64µV). Taken together, the FRN was modulated by the true value but not the face value of money. The FRN effect (losing minus winning) in four experimental conditions and the corresponding topographical maps were shown in Figure 6.

Similar analysis was also conducted for the P300 (Figure 4 and Figure 5 D–F). Repeated-measures ANOVA using price (cheap vs. expensive), magnitude (small vs. large), and feedback valence (loss vs. win) as independent factors found a significant main effect of magnitude, F(1,18) = 7.20, p<0.02 and a significant main effect of feedback valence, F(1,18) = 39.29, p<0.001. There was no significant interactions involving feedback valence, p>0.1, suggesting that the P300 effect (loss minus win) was not modulated by either face value or true value.

## Discussion

Our study demonstrates behavioral money illusion in laboratory using self-reported satisfaction ratings. Participants felt more satisfied for winning large magnitude reward in expensive context than for winning small magnitude reward in cheap context, although the two types of reward have an identical real value. Similarly, they felt more dissatisfied for losing large reward in expensive context than for losing small magnitude reward in cheap context. At the neural level, we found that the FRN, which was believed to be generated in the ACC, was modulated by the true value but not the face value. The P300 was not modulated by either the face value or the true value.

An intriguing question about money illusion is whether the true value has ever been computed in the brain or not. Our findings suggest that the true value is encoded in the ACC (indexed by the FRN) at an early stage. The ACC is anatomically well positioned to integrate reward information given its cortico-cortico, sensorimotor, and subcortical connections [13], [14]. Previous human fMRI studies have shown that the dorsal and rostral areas of the ACC both seem to be affected by rewards and losses associated with errors [15]–[17]. Monkey single-unit recording studies also show that cells in the ACC have an evaluative component, encoding reward values and affective responses to errors [18], [19]. Importantly, previous studies have also shown that the ACC (and also other reward regions) evaluates reward in a context-dependent fashion, being sensitive to the relative reward rather than the absolute value of the reward [20]–[22]. Thus, the ACC plays a crucial role in rapidly extracting the essential reward information in complex contexts.

Our FRN results, however, seems at odds with the previous findings that the magnitude of reward does not affect the FRN [23], [24]. It has been argued that evaluative information processed by the anterior cingulate cortex is simple rather coarse in nature and the FRN only provides a discrete evaluation of events as good or bad regardless of magnitude. However, in our study, the FRN was modulated by the price level context. The FRN effect was larger when price level is low than when price level is high. Other studies have demonstrated that the FRN is influenced by both valence and magnitude [25], [26]. It has also been shown that the FRN precisely reflects the magnitude of reward prediction errors [27], [28], suggesting that the FRN can provide quantitative rather than only qualitative information concerning reward outcomes. Taken together with these studies, our research shows that the FRN is sensitive to fine-tuned reward processing and suggests that the functional significance of the FRN goes beyond simple identification of valence.

Previous research has demonstrated that various aspect of reward values (e.g. action values, relative values, expected values, and experienced values) are represented in different brain areas. Neuroimaging studies have already identified a number of regions that are sensitive to reward magnitude, including the orbitofrontal cortex, insula, and ventral striatum [21], [29]–[32]. Moreover, reward and punishment signals might be broadly distributed in the entire brain [33]. Although the ACC is generally believed to be the main generator of the FRN [8], [34], [35], recent studies combining fMRI and ERP measures of reward processing found that monetary gains elicit the FRN and activate the ventral striatum, medial prefrontal cortex, caudate, amygdala and orbital frontal cortex. Thus, there might be other brain regions (e.g. striatum) that may also generate the FRN [36], [37]. The ACC might not be the only region that encodes the true value of money. A number of other cortical and subcortical regions may also contribute to the processing of true values. Future fMRI studies are needed to further investigate how true value signals and face value signals are separately represented and how they are integrated.

Previous studies have shown that P300 is implicated in a large number of cognitive and affective processes and is traditionally associated with allocation of mental resources. It is often elicited using a simple discrimination task called “oddball paradigm”, in which participants are required to respond to infrequent stimuli presented among a series of frequent stimuli [38]–[41]. In recent years, the P300 effects have also been observed in tasks involving decision making or outcome evaluation [10], [24], [42], [43]. Yeung and Sanfey (2004) found that the P300 was sensitive to the valence of the alternative outcome, with a larger P300 associated with a positive outcome. Our work shows that the P300 is more positive for wins than for losses, which is consistent with previous work highlighting the role of P300 in processing the valence of stimuli [10], [11] However, in the present study, neither the face value nor the true value of money modulate the P300, suggesting that the P300 is only involved in the binary evaluation of outcomes (good vs. bad). However, it also possibly that our manipulation of true value and face value is not powerful enough to elicit difference in the P300, although participants felt enhanced emotional responses to large face value outcomes. Whether the P300 is influenced by reward magnitude is still under debate and need further investigation.

Although all outcomes were predetermined and randomized, it is possible that participants may still actively try to learn associations among cues (some arbitrary features), responses (left/right) and outcomes (win/loss). However, post-experiment debriefing did not identify any performance strategies deliberately used (although unconscious strategies cannot be ruled out) and it is unlikely that the learning would differ between expensive and cheap conditions. Nevertheless, the subjective values of the same outcomes may be modulated by learning (e.g. predictions and prediction errors) and may fluctuate across trials. Future studies may use computational models to quantify the subjective utility of outcomes in each trial more precisely.

In the present study, we used the method of EEG which carries its own advantages to study the value computation. Several previous studies have demonstrated that several early ERP components (e.g. FRN and P300) are modulated by the valence, magnitude and expectancy of reward feedback [11], [44]. For example, Harris et al found that the value signals were represented as early as 150ms after stimulus onset and value-related responses evolved over time across three time windows: 150–250 ms, to 400–550 ms, and 700–800 ms [45]. Source reconstruction using Granger causality revealed that the distribution of value-related activity shifted from posterior to anterior, and from parietal to central to frontal sensors [45], suggesting that different brain regions are engaged at the different phases of reward processing. The EEG recording can offer the ability to measure value computation signals with high temporal resolution without excessive sacrifices in spatial localization. The low temporal resolution of fMRI is ill-suited to examine the temporal dynamics of reward processing during money illusion. Previous behavioral studies have provided evidences to support the existence of money illusion. But the time courses of encoding the face and true value are still unclear. Thanks to the high temporal resolution of EEG, we found that the true value of money was represented within approximately 250 ms after the onset of the feedback information. Although our results demonstrated that the magnitude of true value is already encoded at the early stage, there was no evidence that nominal value modulates reward processing before 250ms.It is possible that the encoding of reward values occurs rapidly at the early stage and money illusion may only occur after this time window. Our study strengthens the connection between the FRN and reward processing and points out the importance of using EEG to study the temporal dynamics of rapid reward processing.

In conclusion, we show that money illusion does exist and can be demonstrated using simple self-reported ratings. Even when robust money illusion occurs, at the neural level, the FRN is modulated by the true value of money, suggesting that the human brain rapidly computes the true value but may ignore such signal subsequently.
